# Supplementary material for: Diversity and Differentiation of Duckweed Species from Israel
Source: Plants (Basel). 2022 Dec 1;11(23):3326. doi: 10.3390/plants11233326 (PMC9736646; doi:10.3390/plants11233326)
Supplement: Supplementary file 1 [file plants-11-03326-s001.zip › plants-1993820-supplementary.pdf]

Supplementary information

# Diversity and Differentiation of Duckweed Species from Israel

**Avital Friedjung Yosef [1,*], Lusine Ghazaryan [1], Linda Klamann [1], Katherine Sarah Kaufman [1], Capucine Baubin [1,2], Ben Poodiack [1], Noya Ran [1], Talia Gabay [1,3], Shoshana Didi-Cohen [4], Manuela Bog [5], Inna Khozin-Goldberg [4] and Osnat Gillor [1,*]**

[1] Zuckerberg Institute for Water Research, J. Blaustein Institutes for Desert Research, Ben Gurion University, Midreshet Ben Gurion 8499000, Israel
[2] Department of Ecology and Evolutionary Biology, University of Colorado Boulder, Boulder, CO 80309, USA
[3] Department of Life Sciences, Ben-Gurion University of the Negev, Be'er Sheva 8410501, Israel
[4] French Associates Institute for Agriculture and Biotechnology of Drylands, The Jacob Blaustein Institutes for Desert Research, Ben-Gurion University of the Negev, Midreshet Ben-Gurion 8499000, Israel
[5] Institute of Botany and Landscape Ecology, University of Greifswald, 17489 Greifswald, Germany
* Correspondence: avitush.f.y@gmail.com (A.F.Y.); gilloro@bgu.ac.il (O.G.)

Supplementary Tables

Table S1. Duckweed plants collected in the present study - detailed location

| Lab Code | Species | Region | loction | Grid Reference | Seasonality of the water source |
|---|---|---|---|---|---|
| 32b | *Lemna gibba* | Golan Hights | "Orvim Reseroim" | 33.137779, 35.725382 | seasonal |
| 58 | | HaSharon | Avihail straem | 32.333731, 34.876507 | perennial |
| 56 | | Golan Hights | E'n Mokesh | 33.09779, 35.81729 | seasonal |
| 7 | | Golan Hights | Natur | 32.867917, 35.770194 | seasonal |
| 43b | | Hulla Vally | Yesud HaMa'ala | 33.060001, 35.615137 | seasonal |
| 43a | | Hulla Vally | Yesud HaMa'ala | 33.060002, 35.615138 | seasonal |
| 31a | | Golan Heights | "Orvim Reseroim" | 33.139682, 35.733806 | perennial |
| 31b | | Golan Heights | "Orvim Reseroim" | 33.139682, 35.733806 | perennial |
| 30 | | Golan Heights | "Orvim Reseroim" | 33.138450, 35.734019 | seasonal |
| 45a | *Lemna minor* | Galille | E'n Korazim | 32.912915, 35.569178 | perennial |
| 42 | | Hulla Vally | Jorden river | 33.015434, 35.629857 | seasonal |
| 44 | | Hulla Vally | Yesud HaMa'ala | 33.064187, 35.610817 | seasonal |
| 57 | | HaSharon | Alexander river | 32.363913, 34.958369 | perennial |
| 45b | | Galille | A E'n Korazim | 32.912915, 35.569178 | perennial |
| 55a | *Lemna minuta* | Golan Heights | Nov stream | 32.801403, 35.783032 | seasonal |
| 43 | | Hulla Vally | Yesud HaMa'ala | 33.060002, 35.615138 | seasonal |
| 55b | | Golan Hights | Nov stream | 32.801403, 35.783032 | seasonal |
| 19 | *Spirodela polyrhiza* | Golan Heights | Keshet | 32.969559, 35.820036 | seasonal |
| 11b | *Wolffia arrhiza* | Golan Heights | Daliyot Junctionl | 32.894030, 35.775695 | seasonal |
| 30a | | Golan Heights | "Orvim Reseroim" | 33.138450, 35.734019 | seasonal |
| 31b | | Golan Heights | "Orvim Reseroim" | 33.139682, 35.733806 | perennial |
| 55 | | Golan Heights | Nov stream | 32.801403, 35.783032 | seasonal |
| 32b | | Golan Heights | "Orvim Reseroim" | 33.137779, 35.725382 | seasonal |
| 30b | | Golan Heights | "Orvim Reseroim" | 33.138451, 35.734018 | seasonal |
| 11a | | Golan Heights | Daliyot Junctionl | 32.895829, 35.776775 | seasonal |
| 58 | *Wolffia globosa* | HaSharon | Avihail stream | 32.333731, 34.876507 | perennial |

**Table S2.** *Water samples analysis:* estimated pH and electric conductivity (EC).

| Lab Code | Species | PH | EC (S m$^{-1}$) |
|---|---|---|---|
| 32b | *Lemna gibba* | 6 | 357 |
| 58 | | 6 | 406 |
| 56 | | 6 | 388 |
| 7 | | 6 | 552 |
| 43b | | 6 | 323 |
| 43a | | 6 | 306 |
| 31a | | 6 | 346 |
| 31b | | 6 | 375 |
| 30b | | 6 | 349 |
| 45a | *Lemna minor* | 6 | 502 |
| 42 | | 5 | 148 |
| 44 | | 6 | 693 |
| 57 | | 6 | 416 |
| 45b | | 6 | 751 |
| 55a | *Lemna minuta* | 6 | 970 |
| 43 | | 6 | 397 |
| 55b | | 6 | 664 |
| 19 | *Spirodela polyrhiza* | 6 | 309 |
| 11b | *Wolffia arrhiza* | 6 | 552 |
| 30a | | 6 | 349 |
| 31b | | 6 | 422 |
| 55 | | 6 | 694 |
| 32b | | 6 | 359 |
| 30b | | 6 | 675 |
| 11a | | 6 | 554 |
| 58 | *Wolffia globosa* | 5 | 412 |

**Table S3.** Full table of fatty acid composition and content of duckweed plants isolates collected in the current study. The strain *W. arrhiza* 9528 was added to the analysis as a reference data continuance.

| **Clone** | **Fatty acid (% of total fatty acids)** | | | | | | | | | | | | | **ω3/ω6 18:3/18:2** | **TFA (%DW)** |
|---|---|---|---|---|---|---|---|---|---|---|---|---|---|---|---|
| | **16:0** | **16:1** | **16:12** | **16:2** | **16:3** | **18:0** | **18:1n9** | **18:1n7** | **18:3n6 GLA** | **18:3n3 ALA** | **18:4n3 SDA** | **20:0** | **22:0** | | |
| *L. gibba* 58 | 20.56±0.43 | 3.91±0.42 | 1.02±0.29 | 2.2±0.05 | 0.52±0.13 | 0.92±0.04 | 1.13±0.13 | 0.59±0.01 | 0.61±0.05 | 52.88±0.65 | 2.62±0.08 | 0.37±0.01 | 0.44±0.04 | 1.13±0.03 | |
| *L. gibba* 30 | 19.72±0.46 | 4.88±0.03 | 0.89±0.01 | 3.27±0.05 | 0.75±0.04 | 1.01±0.16 | 1.07±0.09 | 0.94±0 | 0.89±0.05 | 50.32±0.36 | 3.81±0 | 0.45±0.01 | 0.39±0.02 | 4.77 | 5.25 |
| *L. gibba* 31a | 21.32±0.05 | 4.28±0.04 | 0.75±0.04 | 2.06±0.01 | 0.17±0.02 | 1.25±0.01 | 0.78±0 | 0.61±0.01 | 0.72±0 | 50.26±0 | 4.37±0.03 | 0.53±0 | 0.57±0.01 | 4.81 | 4.39 |
| *L. gibba* 31b | 21.61±0.17 | 5.46±0.31 | 0.87±0 | 2.18±0.02 | 0.23±0.07 | 1.07±0.04 | 1.11±0.01 | 0.56±0.01 | 0.39±0 | 48.98±0.01 | 1.96±0.03 | 0.39±0 | 0.41±0.01 | 4.46 | 3.63 |
| *L. gibba* 32 | 20.01±0.42 | 4.65±1.09 | 0.55±0.04 | 2.11±0.03 | 0.35±0.21 | 1.36±0.05 | 0.94±0.01 | 0.57±0.01 | 0.8±0.01 | 48.09±0.21 | 3.21±0.01 | 0.55±0.02 | 0.51±0.05 | 3.52 | 3.88 |
| *L. gibba* 43a | 20.96±0.23 | 3.69±0.42 | 0.92±0.03 | 2.07±0.06 | 0.65±0.08 | 1.01±0.08 | 1.47±0.24 | 0.78±0.02 | 0.61±0.07 | 49.87±0.91 | 2.06±0.14 | 0.48±0.02 | 0.51±0.01 | 3.13 | 4.10 |
| *L. gibba* 43b | 21.4±0.31 | 4.56±0.77 | 1.01±0.14 | 1.96±0.03 | 0.43±0.13 | 0.97±0.01 | 1.3±0.02 | 0.71±0 | 0.47±0 | 49.92±0.24 | 2.02±0 | 0.39±0.01 | 0.41±0.02 | 3.60 | 4.36 |
| *L. gibba* 56 | 21.75±2.27 | 5.39±1.1 | 0.97±0.11 | 2.62±0.02 | 0.23±0.11 | 1.08±0.31 | 1.08±0.19 | 0.85±0.06 | 0.52±0.11 | 47.77±3.37 | 2.35±0.34 | 0.42±0.14 | 0.45±0.15 | 3.65 | 4.12 |
| *L. gibba* 7 | 21.49±0.04 | 4.23±0.22 | 0.69±0.05 | 2.24±0.06 | 0.4±0.03 | 1.24±0.03 | 0.94±0.01 | 0.72±0.02 | 0.44±0.05 | 47.78±0.51 | 2.1±0.03 | 0.53±0.01 | 0.5±0.01 | 3.49 | 4.09 |
| *L. minor* 42 | 22.73±0.12 | 5.67±0.37 | 0.39±0.01 | 1.62±0.09 | 0.35±0.06 | 1.8±0.12 | 1.13±0.02 | 0.67±0 | 0.46±0.01 | 45.8±0.03 | 1.51±0.14 | 0.55±0 | 0.31±0 | 3.12 | 2.83 |
| *L. minor* 44 | 19.85±0.06 | 5.5±0.01 | 0.33±0 | 2.01±0.01 | 0.2±0.01 | 1.28±0.02 | 1.45±0 | 0.58±0 | 0.5±0 | 46.75±0.13 | 1.75±0 | 0.31±0.01 | 0.29±0.01 | 2.86 | 3.35 |
| *L. minor* 45a | 19.97±0.33 | 2.89±0.05 | 0.31±0.01 | 2.03±0.02 | 0.75±0.02 | 1.4±0.04 | 1.69±0.01 | 0.7±0.03 | 0.6±0.01 | 47.37±0.39 | 1.88±0.08 | 0.47±0.01 | 0.36±0.15 | 2.54 | 6.34 |
| *L.minor* 45b | 22.49±0.9 | 4.1±0.79 | 0.51±0.03 | 2.54±0.43 | 0.68±0.37 | 1.4±0.16 | 1.57±0.03 | 1.11±0.13 | 0.71±0.05 | 42.74±0.63 | 2.68±0.21 | 0.45±0.01 | 0.34±0.14 | 2.51 | 3.77 |
| *L. minor* 57 | 18.28±0.16 | 5.8±0.18 | 0.51±0.02 | 2.77±0.02 | 0.22±0.04 | 0.77±0.02 | 1.33±0.01 | 0.43±0 | 1.27±0.01 | 48.36±0.19 | 3.04±0.01 | 0.17±0.01 | 0.24±0 | 2.42 | 3.52 |
| *L. minuta* 43 | 18.48±0.45 | 4.64±0.03 | 0.28±0 | 2.43±0.05 | 0.52±0.05 | 1.24±0.39 | 1.27±0.02 | 0.43±0.04 | 0.23±0.21 | 54.43±1.13 | 0.09±0.02 | 0.25±0.08 | 0.29±0.05 | 3.00 | 4.19 |
| *L. minuta* 55a | 19.92±0.2 | 4.97±0.52 | 0.37±0 | 1.62±0 | 0.31±0.13 | 0.93±0.03 | 1.08±0.02 | 0.32±0 | 0±0 | 53.35±0.23 | 0.12±0.11 | 0.15±0.02 | 0.19±0.03 | 3.87 | 4.78 |
| *L. minuta* 55b | 20.07±0.12 | 3.44±0.07 | 0.31±0.01 | 1.6±0 | 0.66±0.04 | 1.03±0.02 | 1.35±0.07 | 0.42±0.03 | 0±0 | 53.51±0.09 | 0.13±0.18 | 0.25±0 | 0.15±0 | 3.36 | 3.95 |
| *S. polyrhiza* 19 | 23.15±0.24 | 5.43±0.37 | 0.45±0.01 | 6.11±0.06 | 0.35±0.04 | 2.35±0.07 | 1.17±0.04 | 1.03±0.06 | 0.04±0.05 | 50.9±0.48 | 0±0 | 0.58±0.04 | 0.62±0.01 | 3.36 | 4.19 |
| *W. arrhiza* 11a | 23.22±1.1 | 5.64±0.58 | 0.51±0 | 3.64±0.23 | 0.55±0.03 | 1.62±0 | 2.17±0.08 | 0.57±0.01 | 0.28±0.01 | 34.87±0.64 | 0.1±0.01 | 0.77±0.01 | 0.64±0.04 | 9.73 | 4.27 |
| *W. arrhiza* 11b | 21.63±0.26 | 5.36±0.53 | 0.49±0 | 3.45±0.12 | 0.59±0.11 | 1.48±0.07 | 1.87±0.03 | 0.52±0.03 | 0.15±0.01 | 37.32±0.56 | 0.1±0.01 | 0.73±0.01 | 0.53±0.01 | 1.38 | 4.38 |
| *W. arrhiza* 30 | 20.4±0.01 | 5.32±0.2 | 0.48±0 | 3.46±0.02 | 0.48±0 | 1.17±0.02 | 1.73±0.01 | 0.49±0.01 | 0.14±0 | 42.29±0.14 | 0.08±0 | 0.58±0 | 0.47±0.01 | 1.45 | 4.55 |
| *W. arrhiza* 31 | 24.28±0.14 | 3.11±0.22 | 0.49±0.01 | 3.19±0.14 | 1.03±0.14 | 1.35±0.04 | 1.64±0.04 | 0.72±0.1 | 0.11±0.1 | 38.74±0.33 | 0.14±0.02 | 0.69±0.02 | 0.4±0 | 1.86 | 4.70 |

| | | | | | | | | | | | | | | |
|---|---|---|---|---|---|---|---|---|---|---|---|---|---|---|
| *W. arrhiza* 55 | 21.85±0.43 | 3.65±0.25 | 0.44±0.03 | 3.33±0.04 | 0.75±0.04 | 1.37±0.11 | 1.73±0.04 | 0.53±0.01 | 0.03±0.01 | 38.97±0.44 | 0.1±0.01 | 0.6±0.02 | 0.51±0.04 | 1.63 | 3.18 |
| *W. arrhiza* 9528 | 19.05±0.13 | 3.72±0.46 | 0.32±0.01 | 2.48±0.03 | 0.24±0.02 | 1.81±0.01 | 1.79±0.01 | 0.35±0.01 | 0.14±0.01 | 44.41±0.1 | 0.07±0 | 0.86±0.02 | 0.66±0.05 | 1.52 | 5.13 |
| *W. globosa* 58 | 22.71±0.05 | 4.04±0.58 | 0.76±0.08 | 1.5±0.05 | 0.35±0.08 | 2.25±0 | 2.41±0.01 | 0.61±0.04 | 0.07±0.39 | 39.51±0.04 | 0.02±0.02 | 0.53±0.01 | 0.39±0.02 | 1.83 | 4.44 |
| *W. globossa* Mankai | 21.73±0.16 | 4.39±0.64 | 0.66±0 | 2.34±0.02 | 0.74±0.21 | 2.14±0.02 | 1.9±0.01 | 0.74±0.05 | 0.14±0 | 44.64±0.21 | 0.07±0 | 0.41±0.02 | 0.19±0.01 | 1.58 | 4.38 |

Supplementary figure

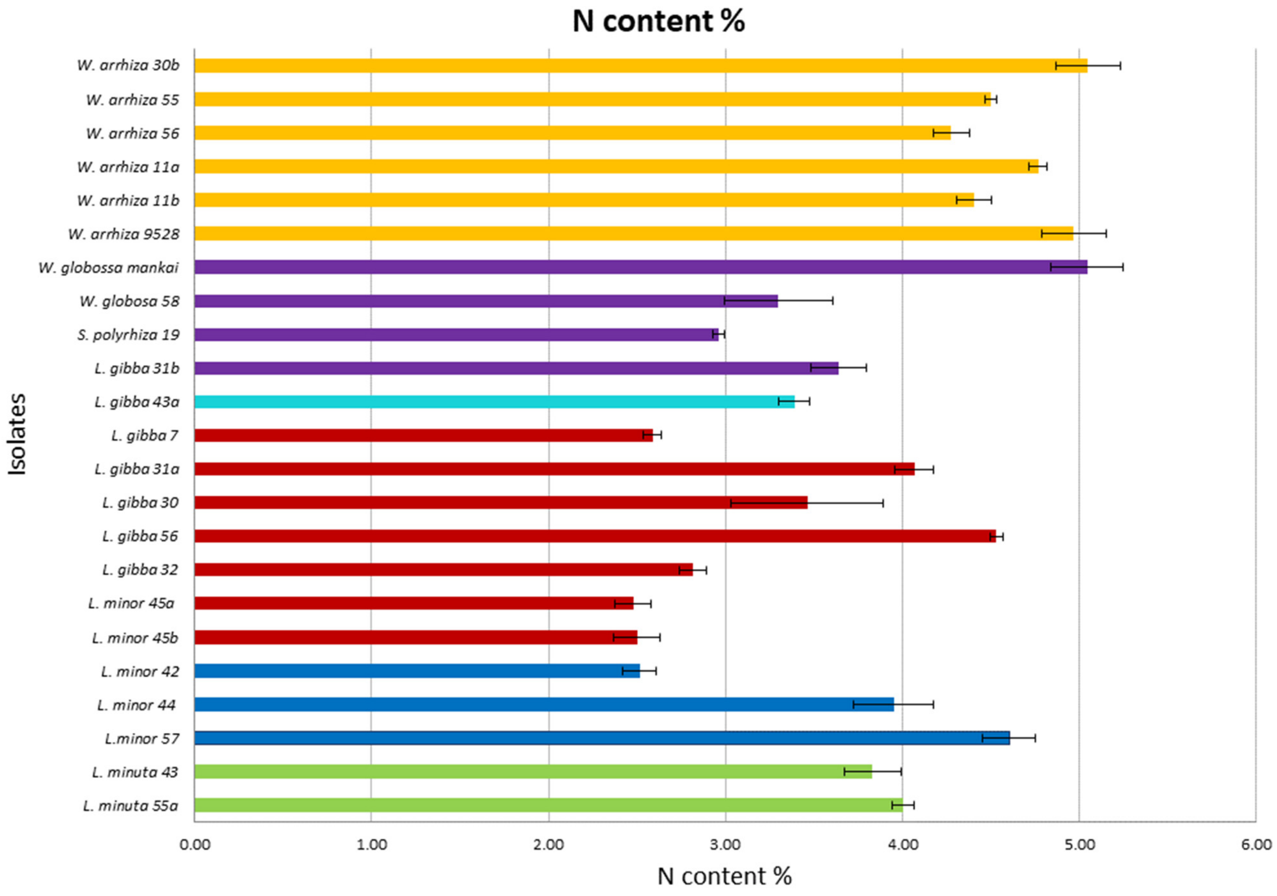


Figure S1. Nitrogen concentration measured by N content FT-MIT.

Supplementary File.

File S1.

*FTIR spectra collection*

The samples were all sieved to pass through a 210 μm mesh. Of each of the sample, three replicate subsamples of approximately 25 mg were scanned. Reference samples of an in-house standard *Lemna* sp. (Ma-STD), and blanks, were measured in each run for quality control. Analysis was conducted with a TENSOR II benchtop FT-IR (Fourier-Transform Infrared) spectrometer (Bruker, Berlin, Germany). This has a spectral range of 8000–340 cm−1, a KBr broadband beam-splitter and window, and an MCT (mercury cadmium telluride) mid-band detector cooled by liquid nitrogen. Diffuse Reflectance Infrared Fourier Transform (DRIFT) spectra were collected. A background spectrum was taken with a gold-plated reference cap. The high throughput screening accessory (HTS-XT), which scans 95 samples in one plate, was used. The spectral resolution was 4 cm−1 and scan time was 32 s per sample. Absorbance data in the spectral range 4000–600 cm−1 were obtained. All the data were obtained and processed using the Bruker OPUS-QUANT II software (Bruker, Berlin, Germany). Corrections of the raw data were made using the first derivative, with 8 smoothing points using the Savitsky–Golay algorithm and mean centred vector normalization. CO2peaks at 2361 and 2339 cm−1 were removed from the data.

Mid-infrared chemometric models were built using PLS (partial least-squares) modelling with OPUS-QUANT II software. A matrix is formed from the spectral data of the calibration samples of known composition. The matrix is transformed by the PLS algorithm into a result matrix consisting of eigenvectors (factors). The predictive reliability of the chemometric model strongly depends on the choice of the rank (the correct number of factors needed). In this case, a Cross Validation (leave one out) system is used to calculate the optimum rank by looking at the root mean square error of prediction (RMSE) with the minimum potential for over-fitting. Assessments of model predictive performance are made with calculations of the correlation coefficient (a measure of relative precision and closeness to the line of best fit), the coefficient of determination ($R^2$, gives the percentage of variance present in the true component values, which is reproduced in the prediction), the RMSECV (root mean squared errors of cross validation), the residual prediction deviation for the rank (RPD = SD/SECV), which allows comparison of model performance across different data sets, and the bias (mean value of deviation, also called "systematic error"). Additionally, the wavenumbers with the highest coefficient explaining the most variation in TN (VIP scores) were identified.
